# Supplementary material for: Why Neurons Have Thousands of Synapses, a Theory of Sequence Memory in Neocortex
Source: Front Neural Circuits. 2016 Mar 30;10:23. doi: 10.3389/fncir.2016.00023 (PMC4811948; doi:10.3389/fncir.2016.00023)
Supplement: Supplementary file 1 [file Table1.PDF]

Supplementary Material

Why Neurons Have Thousands of Synapses, A Theory of Sequence Memory in Neocortex

Jeff Hawkins[1]*, Subutai Ahmad[1]

[1]Numenta, Inc., Redwood City, CA USA

*** Correspondence:** Jeff Hawkins, Numenta Inc., 791 Middlefield Road, Redwood City, CA 94063 USA.
jhawkins@numenta.com

1. S1 Table. Comparison of Common Sequence Memory Algorithms

	HTM	HMMs	LSTM
High order sequences	Yes	Limited	Yes
Discovers high order sequence structure	Yes	No	Yes
Local learning rules	Yes	No	No*
Continuous learning	Yes	No	No
Multiple simultaneous predictions	Yes	No	No
Unsupervised learning	Yes	Yes	No
Robustness and fault tolerance	Very high	No	Yes
Detailed mapping to neuroscience	Yes	No	No
Probabilistic model	No	Yes	No

Table comparing two common sequence memory algorithms (HMM and LSTM) to proposed model (HTM).

* Although weight updated rules are local, LSTMs require computing a global error signal that is then back propagated.
