# Supplementary material for: Why Neurons Have Thousands of Synapses, a Theory of Sequence Memory in Neocortex
Source: Front Neural Circuits. 2016 Mar 30;10:23. doi: 10.3389/fncir.2016.00023 (PMC4811948; doi:10.3389/fncir.2016.00023)
Supplement: Supplementary file 2 [file Image1.PDF]

Supplementary Material

Why Neurons Have Thousands of Synapses, A Theory of Sequence Memory in Neocortex

Jeff Hawkins[1]*, Subutai Ahmad[1]

[1]Numenta, Inc., Redwood City, CA USA

*** Correspondence:** Jeff Hawkins, Numenta Inc., 791 Middlefield Road, Redwood City, CA 94063 USA.
jhawkins@numenta.com

S1 Text. Chance of Error When Recognizing Large Patterns with a Few Synapses

Formula for calculating chance of error

A non-linear dendritic segment can robustly classify a pattern by sub-sampling (forming synapses to) a small number of cells from a large population. Assuming a random distribution of patterns, the exact probability of a false match, following is given by the following equation:

$$\frac{\sum_{b=\theta}^{s} \binom{s}{b} \times \binom{n-s}{a-b}}{\binom{n}{a}}$$

$n =$ cell population size
$a =$ number of active cells
$s =$ number of synapses on segment
$\theta =$ NMDA spike threshold

Table A: Chance of error due to sub-sampling

This table demonstrates the effect of sub-sampling on the probability of a false match using the above equation. The chance of an error drops rapidly as the sampling size increases. A small number of synapses is sufficient for reliable matching.

s	Probability of false match
**6**	9.9×10^{-13}
**8**	9.8×10^{-17}
**10**	9.8×10^{-21}

$n = 200{,}000$
$a = 2{,}000$
$\theta = s$

Table B: Chance of error with addition of 50% noise immunity

This table demonstrates robustness to noise. By forming more synapses than required for an NMDA spike, a neuron can be robust to large amounts of noise and pattern variation and still have low probability of a false match. For example, with $s = 2\theta$ the system will be immune to 50% noise. The chance of an error drops rapidly as θ increases; even with noise a small number of synapses is sufficient for reliable matching.

θ	s	Probability of false match
6	12	8.7×10^{-10}
8	16	1.2×10^{-12}
10	20	1.6×10^{-15}
12	24	2.3×10^{-18}

$n = 200{,}000$
$a = 2{,}000$

Table C: Chance of error with addition of mixing synapses on a dendritic segment

This table demonstrates that mixing synapses for m different patterns on a single dendritic segment will still not cause unacceptable errors. By setting $s = 2m\theta$ we can see how a segment can recognize m independent patterns and still be robust to 50% noise. It is possible to get very high accuracy with larger m by using a slightly higher threshold.

θ	m	s	Probability of false match
10	2	40	6.3×10^{-12}
10	4	80	8.5×10^{-9}
10	6	120	4.2×10^{-7}
15	6	120	1.7×10^{-12}

$n = 200{,}000$
$a = 2{,}000$
